# Qualitative study exploring healthy eating practices and physical activity among adolescent girls in rural South Africa

**DOI:** 10.1186/1471-2431-14-211

**Published:** 2014-08-26

**Authors:** Heather M Sedibe, Kathleen Kahn, Kerstin Edin, Tabitha Gitau, Anneli Ivarsson, Shane A Norris

**Affiliations:** 1 MRC/Wits Developmental Pathways for Health Research Unit, Department of Paediatrics, Faculty of Health Sciences, University of Witwatersrand, 7 York Road, Parktown, Johannesburg, South Africa; 2 University of Limpopo (Medunsa Campus), Faculty of Health Sciences, School of Health Care Sciences, Discipline of Human Nutrition and Dietetics, Limpopo, South Africa; 3 MRC/Wits Rural Public Health and Health Transitions Research Unit (Agincourt), School of Public Health, Faculty of Health Sciences, University of Witwatersrand, Johannesburg, South Africa; 4 Epidemiology and Global Health, Department of Public Health and Clinical Medicine, Umeå University, Umeå, Sweden

**Keywords:** Adolescent, Barriers, Eating, Facilitators, Girls, Healthy, Practices, Physical activity, Pairs, Agincourt

## Abstract

**Background:**

Dietary behaviours and physical activity are modifiable risk factors to address increasing levels of obesity among children and adolescents, and consequently to reduce later cardiovascular and metabolic disease. This paper explores perceptions, attitudes, barriers, and facilitators related to healthy eating and physical activity among adolescent girls in rural South Africa.

**Methods:**

A qualitative study was conducted in the rural Agincourt subdistrict, covered by a health and sociodemographic surveillance system, in Mpumalanga province, South Africa. Semistructured “duo-interviews” were carried out with 11 pairs of adolescent female friends aged 16 to 19 years. Thematic content analysis was used.

**Results:**

The majority of participants considered locally grown and traditional foods, especially fruits and vegetables, to be healthy. Their consumption was limited by availability, and these foods were often sourced from family or neighbourhood gardens. Female caregivers and school meal programmes facilitated healthy eating practices. Most participants believed in the importance of breakfast, even though for the majority, limited food within the household was a barrier to eating breakfast before going to school. The majority cited limited accessibility as a major barrier to healthy eating, and noted the increasing intake of “convenient and less healthy foods”. Girls were aware of the benefits of physical activity and engaged in various physical activities within the home, community, and schools, including household chores, walking long distances to school, traditional dancing, and extramural activities such as netball and soccer.

**Conclusions:**

The findings show widespread knowledge about healthy eating and the benefits of consuming locally grown and traditional food items in a population that is undergoing nutrition transition. Limited access and food availability are strong barriers to healthy eating practices. School meal programmes are an important facilitator of healthy eating, and breakfast provision should be considered as an extension of the meal programme. Walking to school, cultural dance, and extramural activities can be encouraged and thus are useful facilitators for increasing physical activity among rural adolescent girls, where the prevalence of overweight and obesity is increasing.

## Background

The eating habits of children and adolescents are of public health interest globally because of growing evidence relating poor childhood nutrition to obesity and increased risks of type II diabetes, metabolic syndrome, and cardiovascular diseases later in life [1]. The recent rapid increase in the overall prevalence of obesity in children and adolescents indicates that environmental factors, and particularly behaviours linked to diet and physical activity, are central to the causation of obesity [2]. South Africa, as a country in economic and health transition, is facing a triple burden of morbidity and mortality from infectious diseases including HIV/AIDS, noncommunicable diseases (NCDs), and violence and injuries [3]. One result of this transition is the increase in obesity prevalence as a risk factor for NCDs [4]. Other risk factors associated with obesity include high energy density diets, high consumption of sugar-sweetened beverages, large portion sizes, eating patterns (such as meal-skipping), high levels of sedentary behaviour and low levels of physical activity [2]. Recently, the World Health Organization (WHO) conducted a survey of physical activity levels in 51 mainly low- and middle-income countries. Among participants aged 18 to 29 years, the prevalence of inactivity was 13.2% in males and 19.1% in females [5]. In a South Africa-based study conducted among females aged 15 to 55 years, where the rate of obesity was 28.9%, women with lower physical activity were found to be at greatest risk for increased body mass index [6]. In the 2013 South African National Health and Nutrition Examination Survey (SANHANES-1), 50.2% of participants aged 18–24 years of age were reported to be inactive [7].

According to the Youth Risk Behaviour Survey conducted in South Africa in 2002 and 2008, among adolescents aged 13 to 19 years (n = 9224), the combined overweight and obesity prevalence almost doubled in black males (6.9% to 11.5%). Among female participants, the prevalence increased significantly from 30% to 37.6% over the same 6-year period [8]. SANHANES-1 reported that in adolescents aged 15 to 17 years of age, the combined prevalence of overweight and obesity was 27.3% in females and 8.8% in males [7]. The risk of overweight and obese youth becoming overweight adults has been demonstrated in a review study [9], and the tracking of both physical activity and diet between childhood and adulthood has also been confirmed [10].

Evidence from rural Agincourt (Mpumalanga province) in South Africa highlights the high prevalence of overweight and obesity among black African females. The prevalence steadily increased with age, reaching 25% by late adolescence. Central obesity risk (waist circumference cut-offs) also increased with puberty and peaked at 35% by early adulthood in females [11].

There is an impetus to investigate in greater depth the gender differences and environmental factors within households, schools, and the community that contribute to adolescent obesity risk. Among urban females in Soweto, South Africa, we found that both at school and during visits to shopping malls, food was commonly shared and money pooled by friends to make joint food purchases [12]. The majority of participants did not prioritise eating breakfast at home, but purchased vetkoek (fried dumplings made from wheat flour) from vendors before school. Lunchboxes were not commonly brought from home; participants preferred to have spending money to purchase food from the school shop. Kota (a quarter loaf of white bread filled with fried potato chips and ample processed meat or cheese), vetkoek, and snacks (maize crisps) were popular lunch choices because of affordability, convenience, peer influence, and palatability. Respondents reported minimal physically active recreational activities. Barriers to activity were the lack of facilities and concerns about community safety [13]. Little research has explored the perceptions of facilitators of and barriers to healthy eating practices and physical activity within rural South African female adolescents.

The aim of this study was to explore perceptions and attitudes of adolescent girls in rural South Africa regarding healthy eating practices and physical activities, in order to learn about rural and urban similarities and differences, using previous findings from Soweto.

## Methods

### Study setting

This study was conducted in rural Agincourt, a subdistrict of Bushbuckridge, Mpumalanga province, northeast South Africa. The study site lies close to the border with Mozambique, bordering the Kruger National Park conservation area. It provides the foundation for the Rural Public Health and Health Transitions Research Unit of the Medical Research Council (MRC) and University of the Witwatersrand, South Africa (the MRC/Wits-Agincourt Unit). The Agincourt Health and Socio-Demographic Surveillance System (AHDSS) spans an area of 420 km^2^ comprising a subdistrict of 27 villages with traditional and elected leadership. The AHDSS was established in the early 1990s with an initial focus on district health systems development, subdistrict health centre networks and referral systems, and training of clinically oriented primary health care nurses [14,15]. In this region, there are high levels of unemployment (between 40 and 50%) and low income levels. Housing types range from traditional mud structures to brick houses built on plots of farm land that are generally insufficient to support subsistence farming. Consequently, crops grown mostly supplement the family diet [11].

### Study design and data collection

We employed the “duo-interview” method to encourage in-depth discussion [16,17]. This approach has previously been successfully applied in urban Soweto. Eleven duo semistructured qualitative interviews were conducted with participants aged 16 to 19 years of age and their close friends residing within rural Agincourt. A close friend was defined as “Someone of your own age group who you know very well, with whom you meet regularly (i.e. a couple of times a week), are engaged in activities with, hang out and/or chill out with, and with whom you share emotional moments. This can be someone from the same neighbourhood, and not necessarily from the same school.”

The sampling and recruitment for this study was done through the AHDSS. Information about the study was discussed with the volunteers and caregivers during the recruitment process. All participants aged 18 years and older gave informed consent. Written consent was obtained from caregivers for those participants aged less than 18 years. Ethics approval for the survey was provided by the University of the Witwatersrand Human Research Ethics Committee (Medical) (M 090427). The current research has adhered to the guidelines for Qualitative research guidelines (RATS) at outlined on http://www.biomedcentral.com/authors/rats.

Fieldwork was conducted by the study manager (TG) with a field worker and transcriber whose first language was Shangaan (the local vernacular) and who resided within Agincourt. The principal researchers (MHS and KE) trained the field workers, including practice interviews that were conducted to ensure that the field worker was conversant with the interview schedule. The interview guide was piloted on two pairs of friends who were not part of the study population, after which changes were made to make the guide more understandable for study participants [18]. The principal researcher (MHS) offered technical assistance during data collection and quality-controlled the interviews.

The interview guide was designed to explore the following: dietary and physical activity practices, attitudes towards healthy eating and physical activity including barriers and facilitators, understanding of health risks associated with obesity, eating and exercise practices at school and outside school, attitudes towards weight control, body image, cultural beliefs, and family factors. The interview schedule domains were informed by the Triadic Influence on Behaviour Model [19-21], which presumes that the intentions behind certain behaviours derive from three streams of influence: the cultural environment, the social environment, and biological and personality factors. Cultural factors represent the broad macro-environment, including religion and ethnicity. The social environment represents the immediate microenvironment, including influences such as household structure, parenting, peers, community, and factors relating to the physical environmental. Biological and personality factors represent stable intrapersonal influences, originating in inherited dispositions (gender and age) and personality characteristics. The Triadic Influence on Behaviour Model has previously been successfully applied in nutrition research [22,23]. Each interview lasted for approximately 70 minutes and was digitally recorded.

### Data handling and analysis

Debriefing sessions were held daily by researchers after the fieldwork to discuss issues and themes emerging from the interviews and to ensure consistency of question meaning. Preliminary analysis occurred concurrently with the continued administration of interviews to identify emergent sub-themes to be pursued in subsequent interviews. Data saturation was reached by the 11^th^ interview. The 11 recorded interviews were transcribed and translated into English by the field worker. Four of the transcribed interviews were randomly selected for a quality check by an external local bilingual transcriber. The researchers who developed the interview schedule listened and the principal researcher (MHS) read the transcripts horizontally (individually) and vertically (across different transcripts) to identify recurrent themes in the data. A co-researcher (KE) read a sub-sample of the transcripts to cross-validate the coding. Thematic content analysis was used [24] and themes were identified according to questions asked in the interview guide. The study findings are presented using similar domains as per the interview schedule structure.

## Results

### Perceptions related to healthy eating practices

Participants believed that traditional foods—specifically miroho (green leafy vegetables), locally grown legumes, vegetables and nuts—are good for health and that their consumption can prevent and cure illness. Participants’ personal attitudes towards certain food items were influenced by traditional beliefs within their households and the community. Quotes below illustrate perceptions of healthy foods:

“Healthy foods are foods that make you live better. With unhealthy food, you will live, but it is not the same as healthy food: it makes you gain weight and become sick. Like carrots—when you have eaten them, they make your eyes whiter and clean. Beetroot and spinach are very important for the human body because they add blood, and spinach makes you healthy in your body.” (pair 3)

“Healthy foods are vegetables because they don’t have fat and you get vitamins and everything in them, unlike meat. It’s not in meat that we get vitamins and everything. Meat is making us sick but I’ve never heard someone say that she is sick because of eating vegetables—they are not causing illness. Food that we are allergic to, which means it’s unhealthy because it is not good for you, and everything that makes you uncomfortable after eating, I can say is unhealthy” (pair 5)

“Healthy food according to my understanding is food that builds your body and protects you from illness, like vegetables. Unhealthy food is food that doesn’t build our body, like sweets, chocolate, and food with a lot of oil.” (pair 6)

“I think that breakfast is very good. You won’t work without eating and you won’t get power without eating, so you have to take breakfast first to be able to do all your activities.” (pair 3)

More than half of the participants believed that breakfast was the most important meal of the day, based on what they had heard and had been taught in school and at local clinics. Most believed in the benefits of breakfast, although many did not eat breakfast at home owing to limited choices or lack of food.

Some mentioned the consequences of not eating breakfast, such as loss of concentration in class or headaches.

“I didn’t eat today. I’m unable to eat in the morning. I eat at around 12 PM, and it is uncommon that I have breakfast. I think breakfast is healthy, because according to law we must not skip breakfast, but I’m used to it, I don’t eat breakfast, I am fine, I don’t feel hungry, and I don’t have a headache. If I eat breakfast I won’t have my lunch.” (pair 5)

There were also signs of embarrassment. It seemed that some participants did not want to voice an opinion about breakfast, as they laughed when asked about their breakfast practices—they said they just get up in the morning, bathe, and go to school. For some participants, skipping breakfast was a coping mechanism to prevent feeling hungry, because they said that if they ate breakfast they would feel hungry sooner before lunch and would not be able to concentrate in class. Very few participants (two) who reported eating breakfast had more than one option available to them. Pap (a maize-based staple) and tea were the most common options among those who consumed breakfast.

### Factors facilitating healthy eating practices

Most participants associated good health with local home-grown foods. Factors that increased consumption of fruits and vegetables were their taste and the feeling of health experienced after eating a particular fruit or vegetable. Family vegetable gardens, which were located within household yards, in vegetable fields outside household yards, at nearby schools, or out in the open fields, enabled healthy eating. Common vegetables grown were beetroot, tomatoes, and green leafy vegetables such as spinach, lettuce, and miroho. According to participants, female caregivers within households collected edible wild green leaves that grow outside the rainy season to eat with pap. Based on interviews, locally grown vegetables were also sold by community members at affordable prices and neighbours often shared with each other. In the few households that did not have vegetable gardens, participants stated that they sourced vegetables from relatives or friends.

The influence of the female caregiver on the foods families consumed was cited as a major factor in facilitating healthy eating practices within households. Based on data from a majority of interviews, vegetable gardens were mainly cultivated by female caregivers who believed that locally grown vegetables were good for health; they cooked vegetables for their families even if some household members did not like eating them.

The quotes below illustrate factors that facilitate healthy eating practices.

“I feel great and healthy when I have eaten lettuce; I just feel good and it makes me happy. I like to cook food for Sunday. I like cooking and making salads, beetroot, pumpkin, and cabbage. Salads are healthy. Healthy food makes a person’s body always be good, but food that has lots of oil, they say, causes high blood pressure and illness for a person. To eat some is not a problem, but she must have a limit in order not always to eat it. I like mango because it is nice. When it is ripe and you eat it, it tastes good. And lettuce—I like it and everything that is grown in the garden; I just like it.” (pair 4)

“According to youth, they think healthy food is meat, but grannies and our parents think it is vegetables.” (pair 1)

“Old people are afraid to eat food with oil because they say it causes illness. They want you also to cook miroho.” (pair 3)

“I like oranges, and when you have eaten them they are good in the body and make you feel great. Then I fell in love with them.” (pair 8)

Health education messages in clinics, magazines, and church youth gatherings were recognised as encouraging healthy eating practices. Local schools with government-supported meal programmes provided cooked meals such as beans with soup, samp (dried corn kernels that have been stamped and chopped until broken but not as fine as mielie-meal or mielie rice) with beans, or tihove, a traditional dish consisting of boiled samp with locally grown crushed nuts. These also served as facilitators of healthy eating practices.

“Everywhere, like when we are in a place that is crowded like the clinic, they teach people that we must eat healthy food in order to help our bodies.” (pair 5)

“When we attend church conferences, they give us carrots, beetroot, cabbage, and a small portion of meat; they also add pumpkin and porridge or rice.” (pair 3)

“At school, we get free healthy food during break. Monday we get pap, Tuesday we get samp, Wednesday rice with soup, Thursday samp with beans, and Friday we get pap with soup or beans.” (pair 6)

### Factors acting as barriers to healthy eating practices

Factors cited as barriers to healthy eating practices were household poverty, the affordability and accessibility of healthier food, peer influence, and aspirations to purchase more socially desirable convenient fast foods. According to the participants, most households do their grocery shopping once a month when they receive money from family members who work in cities far from home. Limited money and transportation means households only purchase basic necessities once a month, including mealie-meal (a maize-based staple), chicken feet, and frozen chicken. Most of the girls mentioned strict grocery lists to which households stick. Groceries purchased monthly often ran out sometime during the month, after which families could only afford to eat pap and miroho that they bought or picked from the fields, as they would have to wait for the end of the next month to purchase more groceries. Eating home-grown vegetables is believed to be a sign of poverty or lack of food, while meat is a sign of wealth or civilisation. Fruits were often cited as “luxuries” or “extras” and were bought only if there was money left after purchasing staple foods. It appeared that fruits were not easily accessible within the community.

“They think it is a sign of better status when eating meat every day.” (pair 5)

“My family doesn’t like miroho and vegetables from the garden, we just like meat and anything from the fridge. When we eat vegetables, we only eat salads and it is not every day that we grow them. They are very scarce.” (pair 1)

Some participants said that they could not bring lunch boxes to school because of limited household resources. They mentioned food items they wished were available for lunch boxes, such as bread, polony (processed deli meat), “Russian” (processed sausages), eggs, “everything that tastes good”, and juice. For those who took lunch boxes to school, the choice was limited to what was available at home. Because of limited lunch money and resources at home, in most cases they were only able to take dry bread augmented with atchar (a pickle made with unripe mangoes and chillies, prepared in oil) or buy vetkoek, because it is affordable.

Some respondents brought lunch money that they stated was insufficient to purchase options that they perceived as relatively healthy, which resulted in them buying cheaper snacks from school vendors. Among items sold by vendors outside the school gate, the majority of participants mentioned bread, vetkoek, kota (a quarter loaf of white bread filled with fried potato chips and ample processed meat or cheese), deep fried potato chips, snacks such as crisps and sweets, sugar-sweetened beverages, atchar, and plates of food with pap and chicken or beef. Based on the interviews, few vendors outside schools sold fruit, which was generally more expensive than snacks—this absence is a barrier to healthy eating. Most participants shared money and food with friends just to make sure they have something to eat.

“I don’t feel good about the free food we get at school, because they don’t cook well. After eating it, I have stomach cramps, so we decided to stop eating the free food at school. If we don’t have money for lunch, we just walk around the schoolyard until lunch is over; if we have some money we buy vetkoek and niknaks (from vendors). We like junk food because we don’t have enough money to make our stomachs full. I don’t like vegetables, I just eat, even if they are healthy, I don’t care about that. When it comes to carrots, I don’t get the taste of it.” (pair 1)

“Usually I take lunch money. When I use it, I buy some snacks and iced lollipops. If we don’t get food at school, I buy kota, niknaks, and vetkoek (sold by school vendors).” (pair 5)

“I like kota when it has everything on it: bread, Russian, cheese, chips, and atchar.” (pair 10)

Peer perceptions were also a barrier to healthy eating. Participants mentioned concerns about their peers’ reactions if they ate miroho, since frequently eating meat or fast food items is seen as a sign of better economic status.

### Perceptions related to physical activity

The majority of participants believed that physical exercise promotes good health, because exercise boosts the body’s ability to fight against illnesses and helps to prevent illness. Even respondents who did not participate in physical activities stated that physical activity is good for health.

“It’s good to exercise. If you exercise, you could lose weight, and it is necessary that every person exercise. At school I’m in athletics and netball. Just now we are writing exams, but [previously] I was always exercising. When I exercise, I’m not lazy and my body is always right; I don’t get the flu easily.” (pair 5)

“I think to exercise is good, but I don’t do it. I’m unable to run or jump, but when my friend says we must do it, I try to do it.” (pair 10)

“Young people should exercise so that the illnesses that are common nowadays cannot get us soon.” (pair 3)

### Practices and factors facilitating physical activity

Most of the schools have a variety of physical activities during school breaks, after school, and during life orientation classes. Most students participate in games such as skipping rope; street dancing; sporting activities including netball, soccer, and volleyball; and a variety of traditional dances. There appears to be positive peer influence promoting physical activity, with active encouragement by friends.

“We like dancing and singing. We play songs on our cell phones and then we dance. Sometimes we just play with kids on the street; we play netball and skip rope.” (*pair 3*)

“It is good because after playing ball, my friend wants to sit down, saying that she is tired. Then I force her, and I set up the clock so that now we will play for twenty minutes—after ten minutes she will play for the whole time we have set.” (pair 10)

Some students walk long distances to and from school, and thus get an opportunity to exercise. At home, most participants were involved in physical household chores such as cleaning, cooking, and working in the vegetable garden or the fields.

“We walk when we go to school. It takes me twenty minutes when I walk fast and forty minutes when I walk slow. I also run, in order to always feel good in the body. During break, we dance the kwaito dance, and we play netball. After school we have netball, ladies soccer, and volleyball. We play netball. When it comes to cultural dances, we have muchongolo, xibavhana, and xipenede [different types of local cultural dances]. We also clean our classrooms after school; then we come home. When we get home, we wash dishes and clean the house.” (pair 6)

### Factors acting as barriers to physical activity

Some participants mentioned that in more senior grades, the school discouraged them from participating in extramural activities. They were encouraged to use that time for studying instead, as sports would disturb them. Most of these participants were involved in sports in junior grades.

“They don’t allow us to play netball or any sports. When you are in grade 12, you don’t participate in anything. Even singing they don’t allow us. They don’t allow it because it will disturb us. This year we are doing nothing at all—like when they[learners in lower grades] go to soccer, we used to go with them just to support them. After school we used to participate in Sarafina dance last year; this year we did nothing at all.” (pair 3)

Despite peer encouragement, a barrier to exercise was peer gossip. Many girls expressed concerns about how they looked when exercising and what their female and male peers would say about them.

“At school there is netball, soccer, ladies soccer, and volleyball. I don’t participate in any activity. My problem is that people who are playing ball at school are talking a lot, and I don’t like to talk.” (pair 8)

## Discussion

Within a rural South African setting, adolescent girls could articulate an understanding of healthy eating. They were aware of healthy versus unhealthy foods, and the benefits of locally grown foods. Most study participants associated healthy foods with health benefits such as prevention of illness and feelings of wellness. Similar perceptions about healthy foods were shared by young females in an urban setting in Soweto, where we have previously investigated the meaning of healthy eating [13]. In both settings, participants described healthy eating in terms of specific foods—in particular, fruits and vegetables, and the benefits of eating these foods, such as improved immune system function and protection from illness. In the current study, the participants described healthy foods as having less fat and including traditional and locally grown foods. The knowledge of health benefits attached to traditional foods imparted by female caregivers and their involvement in household agriculture and food preparation were important factors enabling adolescent girls to eat more healthily. A strong facilitator of healthy eating at the household level was the availability of family-grown vegetables within households or from neighbours, relatives, or local vendors. Interestingly, participants generally did not view the availability of miroho as facilitating healthy eating, but rather as a sign of poverty.

Poverty and food insecurity are factors that are barriers to healthier eating. For a majority of participants, unavailability of food for breakfast at home meant their not eating anything before going to school. For the few who did eat something, pap with tea was most common. Most young women felt that they did not have the resources to eat a healthy diet because of limited choices and restricted access to healthy foods. Given these findings, students may benefit from breakfast programmes such as the Maryland Meals program for Achievement, which provides free breakfast in classrooms. This is currently not common practice in South African government-supported high schools. This approach, where breakfast was supplied in the classroom as part of the school day, caused improvements in performance, attendance, attention, and behaviour [25]. It will play a major role in facilitating healthy eating practices in a community that is reported to have increased household food insecurity due a high prevalence of HIV/AIDS [26].

However, it appears that peer pressure and cultural beliefs may hinder the consumption of traditional foods, as eating miroho is considered a sign of poverty. There is a strong aspiration to consume more meat and fast foods, because they are associated with better economic status and are therefore more desirable. With the benefits of poverty reduction that economic transition brings to South African urban and rural settings [27], it is concerning that healthy traditional and local eating practices could erode as communities adopt unhealthy eating behaviours.

The school meal programme provides cooked meals to school students who otherwise would not have had any food. However, adolescents mentioned that fruit was rarely available, and that the meals served might not be the “healthiest”, with reports of stomach cramps. Increasing the availability of healthy foods through the school meal programme or reduced/subsidised food prices would facilitate healthy eating. This is supported by findings of a systematic review of United Kingdom-based studies examining barriers to and facilitators of healthy eating among young people aged 11 to 16 years. Adolescents overall believed that greater availability of healthy foods would facilitate healthy eating [28]. However, in the current study, despite Agincourt participants acknowledging the school meal programme, they expressed a strong desire to have the financial resources to purchase convenience foods such as fried chips and sugar-sweetened beverages from school vendors. These findings are in line with the systematic review conducted by J. Shepherd et al. in 2006, where young people mostly preferred fast food for its taste and for the ability to choose what they ate [28].

As rural communities transition and become more urbanised, it is important that lessons from urban areas are acknowledged. In a similar study conducted in an urban setting, Soweto girls were skipping breakfast at home and consuming it at school, where school vendors sold unhealthy high-energy options such as vetkoek and snacks instead. Compared with their rural counterparts, urban girls reported consuming more fast foods at home during weekends (such as kota and vetkoek for breakfast) owing to its greater accessibility, convenience, and cost. Some urban girls even replaced supper during the week with kota outside the home, which resulted in reduced sharing of family meals. In rural settings, because of the increasing cost of living, economic challenges, and increasing availability, access, and popularity of fast foods, it is possible that adolescents will consume more fast food. This could cause a decline in the consumption of locally grown and traditional vegetables among adolescents.

It is important to consider the impact of poverty and food insecurity, the importance of informal food vendors in rural communities, the food composition of school meal programmes, and the aspirations of youth (including taste preferences and the emotional connotations of food) when envisaging interventions to promote healthier dietary behaviours. Clinics were also reported to provide health education messages that promote and encourage healthy eating practices.

While urban girls in the Soweto study also participated in house chores, the majority did not walk long distances to and from school, as did their counterparts in the current study, and some even used transportation [13]. In both settings, dancing (street and traditional) can be employed in interventions to increase physical activity. These findings are in line with a study conducted in rural Limpopo province, Dikgale village, where adult women were found to be highly active because they walked with increased intensity for long distances owing to transport limitations, and participated in household work, yard work, and farming activities [29]. In a United States study of Florida adolescents aged 13 to 14 years, walking to school was associated with greater overall levels of vigorous physical activity throughout the day compared with travelling by car, bus, or train [30].

Schools play a major role in facilitating and promoting physical activity among female students. However, schools need to encourage older adolescent learners at higher grades to participate in physical activity in order to encourage ongoing activity after they leave school. Based on recent findings in the same community, increased resources through innovative local organisations such as schools should assist in prioritising the provision of equipment and facilities for non-classroom activities [31]. In a South African township-based study among secondary school students in Durban, inadequate sports facilities were cited as the primary reason for nonparticipation in sports by black students [32].

## Conclusions

The findings of this study will help to formulate strategies to address barriers and build on known facilitators of healthy practices among female adolescents in rural areas, thereby creating conditions that encourage healthy eating practices and physical activities.

As the nutrition transition advances in rural South African settings, it is necessary to protect and promote the availability of and access to locally grown foods and traditional dishes, in order to encourage healthy eating among female adolescents. Female caregivers and the elderly in the community can play an important role in teaching young females about the health benefits of traditional foods, because they are primarily involved in preparing family meals. Food availability in relation to food poverty needs to be addressed. This is a major barrier, because adolescents know about the benefits and importance of consuming breakfast. School meal programmes should be expanded and improved as a contribution to healthy eating among adolescents who do not have sufficient access to healthy options at home.

Physical activities that adolescents currently engage in, such as household chores, walking long distances, and traditional dancing, should be preserved and encouraged in a society with an increasing prevalence of overweight and obesity. Extramural activities at school should be promoted, and sports facilities strengthened. Future studies should explore how other community-based structures such as churches and clinics can be employed to promote and protect healthy eating practices among adolescents. These interventions are vital to help reduce the prevalence of overweight and obesity among young girls and thus reduce the risk of future cardiovascular and metabolic disease.

## Competing interests

The authors declare that they have no competing interests.

## Authors’ contributions

MHS, SN, KK, and KE were involved in the initial conceptualisation of the research question and interview guide. MHS and TG collected the data. MHS coded the data with assistance from KK and SN. MHS was responsible for the data analysis, with input from KE, AI, SN, KK, and AI. MHS took the lead in drafting the manuscript, with input from SN, KE, AI, TG, and KK. All authors read and approved the final manuscript.

## Pre-publication history

The pre-publication history for this paper can be accessed here:

http://www.biomedcentral.com/1471-2431/14/211/prepub
